# Oxidative-Antioxidant Imbalance and Impaired Glucose Metabolism in Schizophrenia

**DOI:** 10.3390/biom10030384

**Published:** 2020-03-02

**Authors:** Amira Bryll, Justyna Skrzypek, Wirginia Krzyściak, Maja Szelągowska, Natalia Śmierciak, Tamas Kozicz, Tadeusz Popiela

**Affiliations:** 1 Department of Radiology, Jagiellonian University Medical College, Kopernika 19, 31-501 Krakow, Poland; amira.bryll@uj.edu.pl (A.B.); msjpopie@cyf-kr.edu.pl (T.P.); 2 Department of Medical Diagnostics, Jagiellonian University, Medical College, Medyczna 9, 30-688 Krakow, Poland; justynaskrzypek96@gmail.com (J.S.); maja.sz@op.pl (M.S.); 3 Department of Child and Adolescent Psychiatry, Faculty of Medicine, Jagiellonian University, Medical College, Kopernika 21a, 31-501 Krakow, Poland; natalia.smierciak@uj.edu.pl; 4 Department of Clinical Genomics, Center for Individualized Medicine, Mayo Clinic, Rochester, MN 55905, USA; kozicz.tamas@mayo.edu

**Keywords:** schizophrenia, reactive oxygen species, glucose metabolism, mitochondrial dysfunction

## Abstract

Schizophrenia is a neurodevelopmental disorder featuring chronic, complex neuropsychiatric features. The etiology and pathogenesis of schizophrenia are not fully understood. Oxidative-antioxidant imbalance is a potential determinant of schizophrenia. Oxidative, nitrosative, or sulfuric damage to enzymes of glycolysis and tricarboxylic acid cycle, as well as calcium transport and ATP biosynthesis might cause impaired bioenergetics function in the brain. This could explain the initial symptoms, such as the first psychotic episode and mild cognitive impairment. Another concept of the etiopathogenesis of schizophrenia is associated with impaired glucose metabolism and insulin resistance with the activation of the mTOR mitochondrial pathway, which may contribute to impaired neuronal development. Consequently, cognitive processes requiring ATP are compromised and dysfunctions in synaptic transmission lead to neuronal death, preceding changes in key brain areas. This review summarizes the role and mutual interactions of oxidative damage and impaired glucose metabolism as key factors affecting metabolic complications in schizophrenia. These observations may be a premise for novel potential therapeutic targets that will delay not only the onset of first symptoms but also the progression of schizophrenia and its complications.

## 1. Oxidative Stress, Impaired Glucose Metabolism, Schizophrenia

Schizophrenia is characterized by a significant reduction of both white and gray matter volume of the brain [1], different levels of anatomical impairment, dysfunction of mitochondrial metabolism, and specific topological patterns [2,3,4], especially in the second decade of individual life. This is confirmed by the computed tomography (CT) and magnetic resonance (MRI) imaging, including the functional magnetic resonance (fMRI) method [5,6,7]. Although the trajectory of the indicated changes remains unexplained, there are reports suggesting their association with early reduction of the volume of the prefrontal cortex, superior temporal gyrus, and hippocampal gyrus volume reduction [8,9]. The probable mechanism of the observed changes in psychosis may be associated with inflammatory disorders, dysregulation of the immune system [3,10], neurodevelopmental disorders, including genetic abnormalities, i.e., 22q11.2 (22q11DS) deletion [11,12], or neutrophil functioning in the severe forms of mental disorders [10]. Their higher levels strongly correlate with positive clinical symptoms, constituting a promising marker of disease progression [13]. In turn, the mentioned genetic disorders (22q11DS) are the cause of the limited growth of axons and dendrites, which impair the integrity of mitochondria and functioning of synapses in productive neurons [11]. This is an important factor linking these changes with trauma, stress, and an increased risk of psychosis among adolescents [12].

The course of schizophrenic disorders can be continuous or episodic, with progressive or permanent cognitive deficits [14]. The first phase (although different in both genders in terms of anatomical differences in the white matter of the brain) is similar in most patients and is characterized by prodromal symptoms: Anxiety, irritability, social withdrawal, and depressed mood [15,16]. Recent evidence-based data also indicate a strong association between schizophrenia and bipolar disorder (BD) through a clear relationship between mitochondrial genes in both diseases [17]. Patients may also experience positive symptoms, i.e., hallucinations and delusions [18]. A late period characterized by stabilization is a chronic disease with numerous relapses and significant functional disorders [19].

The first psychotic episode (FEP) is the earliest stage of the disease, in which patients exhibit motivation deficits and impaired decision making related to cognitive impairment. The decreased desire to put more effort into cognitive functions is caused by the inability to appreciate the reward or overestimating the amount of effort that should be put into a given activity [20]. Patients with FEP also have reduced adaptability due to the brain defects in the hippocampus [21]. The difference between them, and diagnosed schizophrenics, is a shorter history of the disease and/or lack of previous pharmacotherapy. During the postmortem examination, the brains of people with FEP show evidence of significant neuropathology involving the enlargement of lateral ventricles, third ventricle, and brain sulci and fissures. Magnetic resonance imaging (MRI and fMRI) of people with the developing disease in the prodromal phase shows an enlargement of the right inferior frontal gyrus [8,22], that directly correlates with reduced neuronal activity in that region. Changes in brain activity are also observed in regions that include cerebellum, temporal and frontal gyri [23], as well as Heschl’s gyrus, which is also reduced in people with schizophrenia [24,25]. There are also functional changes in the region of the insula and amygdala. Those deficits, disturbing neural communication, are clinically manifested through speech and the disorders of information interpretation, auditory hallucinations, mood disorders, personality turmoil, and depression [26,27]. A decrease in brain bioelectrical activity in the inferior left crescent lobe of cerebellum may be associated with negative symptoms [28] in those patients. In addition, loss of synapses plasticity and impaired electrical conductivity can be observed, which causes elongation of the nerve impulse path and ultimately the death of neurons [29]. These factors attribute to the clinical phenotype of the disease, in which, apart from mental and behavioral problems, cognitive disorders occur, including distortions of thinking and perception, inability to adapt behavior to the situation, or dementia [30], which successively affect executive functions [31]. Depending on the time of diagnosis, early introduction of appropriate pharmacotherapy or psychotherapy of patients and their families guarantees complete recovery after the onset of the first psychotic episode, while its lack becomes the cause of FEP progression into a chronic and full-blown schizophrenia [32] with a worse treatment prediction [33] and is associated with a reduction in the chances of patient’s full recovery [34,35].

Pharmacotherapy of schizophrenia faces some problems including polypragmasia resulting from the lack of standards for the use of polypharmacotherapy [36]. According to the new recommendations of the American Psychiatric Association of 2020, schizophrenia therapy, especially in younger people experiencing the first psychotic episode, should be started with atypical drugs, initially at a low dose, with observation of the response to treatment and side effects [37]. Olanzapine is one of the often chosen drugs with minor side effects from the extrapyramidal system (in contrast to classical neuroleptics). Despite initial hopes, it has not become an “ideal” antipsychotic due to the fact that its long-term use is fraught with a number of cardiometabolic complications, i.e., obesity [38], dyslipidemia, hyperglycemia, insulin resistance, and diabetes [39]. Its mechanism of action is based, among others, on increasing the amount of fatty acid binding protein (FABP4) and reducing the amount of apolipoprotein A-IV [40], which appears to be crucial in the intercellular transport of lipids. This results in a decrease in insulin-dependent translocation of membrane glucose transporters (GLUT4) leading to a decrease in blood glucose uptake [41]. In addition, it is important that the complication of the assessment of risk related to cardiometabolic syndrome is due to some FEP patients showing impaired glucose metabolism preceding the use of antipsychotic treatment [42]. This should be taken into account at the stage of qualifying patients for treatment and during atypical antipsychotics pharmacotherapy based on recommended dose reduction (“doses must be reduced gradually at a rate of approximately 20% every 6 months”) [43] as a risk-benefit strategy prior to treatment [37].

**Figure 1:**
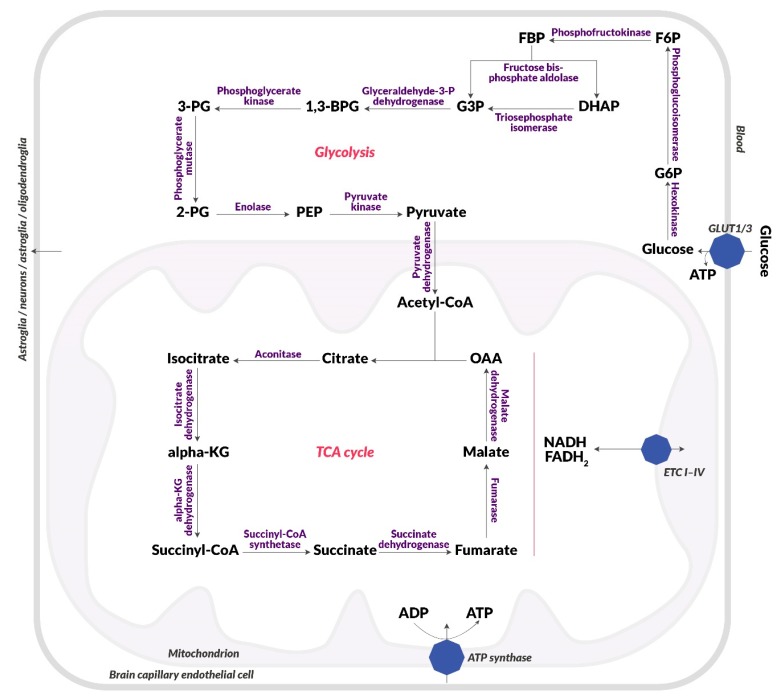
Diagram of glucose metabolism. Blood glucose uptake, glycolysis, citric acid cycle, electron Table 1, and an insulin-independent transporter (GLUT3). Then, it is metabolized in cytosol in glycolysis to pyruvic acid, which passes into the mitochondria. Pyruvate dehydrogenase converts it to acetyl-CoA, which is included in the citric acid cycle (TCA), which runs in the mitochondrial matrix. There, NADH and FADH2 are produced, which end up in the electron transport chain (I-IV) (ETC I-IV), which is located on the inner membrane of the mitochondrion. NADH and FADH2 are used to create a proton gradient, which is then used to produce ATP by ATP synthase. Abbreviations: 1,3-BPG: 1,3-bisphosphoglyceric acid; 2-PG: 2-phosphoglyceric acid; 3-PG: 3-phosphoglyceric acid; α-KG: Alpha-ketoglutarate; F6P: Fructose-6-phosphate; FBP: Fructose-1,6-bisphosphate; G3P: Glyceraldehyde-3-phosphate; G6P: Glucose-6-phosphate; GLUT1: Insulin-independent glucose transporter; GLUT4: Insulin-dependent glucose transporter; IR: Insulin receptor; OAA: Oxaloacetic acid; PEP: Phosphoenolpyruvic acid.

**Figure 2:**
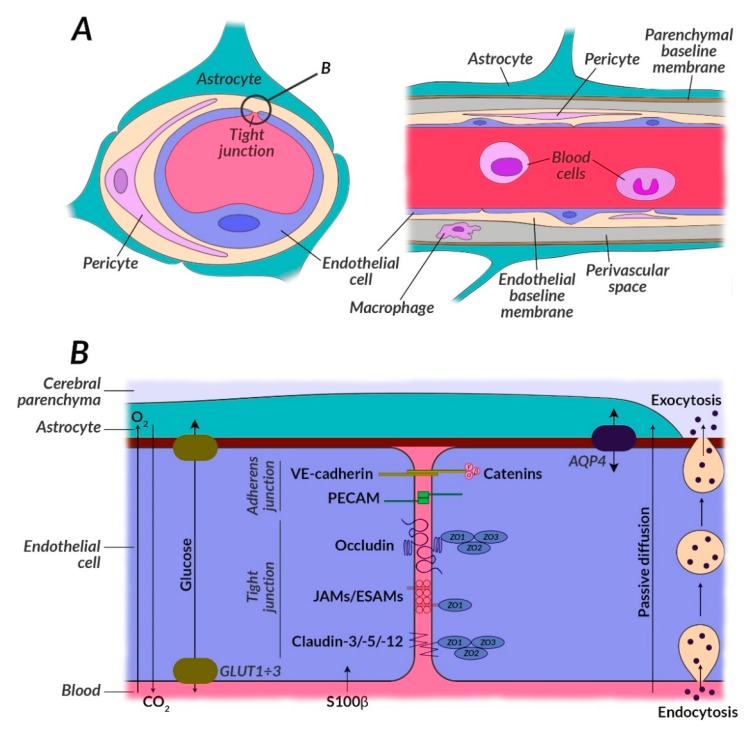
Structure of the neurovascular unit (NVU) with particular attention to the glucose metabolism impairment. (**A**) NVU contains the blood-brain barrier (BBB) which consists of many different cells including astrocytes, pericytes, neurons, microglia, and endothelial cells. The brain cells can work with each other when metabolic demand on glucose and other substrates is enough. BBB provides crossing of important substrates for mitochondrial metabolism, e.g., glucose, removal of toxic superoxides, e.g., carbon dioxide, and reduce access to the brain of potentially damaging molecules in the bloodstream, e.g., some drugs. BBB constitutes a protection against harmful factors due to the mechanisms of active intercellular transport or control by proteins transporting energy substrates located in the vascular endothelial cell membrane (e.g., GLUT1). (**B**) Intracellular transport is regulated by protein complexes located between endothelial cells, i.e., ESAM, AQP4, GLUT1, PECAM, JAMs, VE-cadherin, peripheral proteins of tight junctions (ZO1÷3), claudine (3, 5, 12), and occludin. GLUT3 is seen in neurons, and GLUT1 in endothelial cells; GLUT2 is a glucose sensor present in the hypothalamus in the brain. In the course of schizophrenia, the GLUT1 translocation process, and thus glucose transport, is impaired, among others due to the effect of harmful oxidative stress, inappropriate mitochondrial metabolism, and impaired mTOR pathway. BBB also regulates the exchange of other components, e.g., calcium, elements of the immune system, or indicators of neurobiochemical focal lesions, i.e., S100β protein. Tight connections in the brains of schizophrenic patients can be broken due to the presence of claudin-5-deficient areas. Their expression is reduced in the prefrontal cortex and they are absent along the blood vessels, which may be associated with the penetration of S100β through BBB and, consequently, with a disorder of brain homeostasis. Abbreviations: AQP4: Aquaporin 4; BBB: Blood-brain barrier; ESAM: Endothelial cell-selective adhesion molecule; GLUT1: Insulin-independent glucose transporter; JAM: Junctional adhesion molecule; NVU: Neurovascular unit; PECAM: Platelet-endothelial cell adhesion molecule; VE-cadherin: Vascular endothelial cadherin; ZO1÷3: (**B**) Peripheral proteins of tight junctions (zonula occludens).

One of the key processes that becomes impaired in schizophrenia and its first symptoms is glucose metabolism. Studies confirm the occurrence of disorders of glucose homeostasis at the early stages of the disease, such as elevated fasting glucose and insulin, and reduced glucose tolerance [44]. This dysfunction may be associated with neuronal insulin resistance [45]. Glucose is the primary source of energy for the brain and is metabolized to ATP during glycolysis, the electron transport chain (ETC), and included in the tricarboxylic acid cycle (TCA) as presented in Figure 1. Glucose enters the brain from the vascular system through highly efficient GLUT1 and GLUT4 glucose transporters (Figure 2) [46].

Blocking glucose transporting proteins (GLUT) both centrally and peripherally is the problem of currently used pharmacotherapy of schizophrenia. An important consequence of this is hyperglycemia and the development of metabolic syndrome. Out of the well-known antipsychotic drugs, clozapine blocks GLUT proteins most strongly. Hence, GLUT1 and GLUT3 in the brain are impaired, causing glucose-malnutrition of neurons. This also prunes neurons, therefore negative symptoms prevail in patients with schizophrenia, or relapses, associated with positive symptoms and ineffective neuronal pruning, may occur. Blocking GLUT protein receptors may seem promising and may target the side effects of antipsychotic drugs while inhibiting neuronal pruning due to disabling already damaged neurons and their conduction pathways [47].

Glucose transporters such as GLUT1 and GLUT3, are important because they participate in glucose uptake into both glial cells and neurons. They are not regulated with insulin, but are activated by systemic hypoglycemia, which stimulates glucose receptors located mainly in hypothalamus (including GLUT1 and GLUT3) [48]. The situation is different in the case of GLUT1 and GLUT4 transporters, which are located mainly on the perimeter. In the state of hunger, the GLUT1/GLUT4 ratio increases and glucose is transported to the brain, whose glucose demand is higher than in the periphery [49]. The brain gets glucose using GLUT1 and GLUT3 transporters, which work so fast that they reduce the subsequent steps involved in glucose metabolism in the brain. GLUT1 and GLUT3 are immediately attached to cell membranes. In contrast to these transporters, GLUT4 located in intracellular vesicles on the periphery through hypoglycemia trigger the hunger mechanism coupled with food acquisition by stimulating insulin release, so that energy and body mass remain in balance [50]. Transporters such as GLUT1 and GLUT3 occurring mainly in the frontal lobes are therefore decisive in the brain glucose metabolism in humans.

Modern theories slightly differ from the classical explanations, and assume that in schizophrenic patients, absorption of glucose into brain cells is impaired. That can be associated, (among others), with the wrong concentration of glucose transporters, i.e., GLUT1 and GLUT3, or neuronal insulin resistance [45]. Our preliminary studies on a group of 40 patients with the first episode of psychosis (manuscript in preparation) with the use of magnetic resonance spectroscopy (MRS) showed, that the strongest relationship between the metabolites, i.e., GLU + GLN + GSH 3.7 with positive symptom P scale was observed in a cingulate cortex. We observed that the higher the value of this ratio, the higher the *p* scale score (0.38; *p* = 0.04, Spearman correlation). This suggests that in the group of patients with first episode of psychosis, the symptoms of disease get worse, when the brain has insufficient glucose, in occurrence of intracellular hypoglycemia. That increases with insulin resistance of brain tissues and inefficient glucose transport, which causes an increase of acute disease symptoms, such as confusion, misinterpretation of reality, anxiety, and irritability, i.e., characteristics of the prodrome and the first episode of psychosis [51]. These results, as well as the presented review, indicate the direction of further studies on neuronal insulin resistance in young patients with FEP. The observed changes in insulin signaling associated with impaired glucose metabolism in the brain of patients may be of clinical significance, as suggested by their strong association with higher P scale results according to the PANSS clinical evaluation.

Interestingly, in the period of rapid neuronal growth in fetal life and late childhood, intracellular hypoglycemia causes the transition of symptoms into chronic deficits, which are manifested by a lack of motivation and a desire to learn, as well as withdrawal of social functions or weakening of interpersonal communication, which ultimately exacerbates the symptoms and causes the disease to turn into a chronic form [5,7].

The consequences of this condition affect astrocytes and neurons in the brain, also impairing the functioning of the glutamine pathway, thereby reducing the availability of glutamate to NMDA receptors. Results of our imaging studies support the hypotheses mentioned above, because patients with FEP presented a significantly lower mean value of glutamate GLN 2.45 compared to the control group (*p* = 0.001), and compared to a group of patients with multiform behavioral and personality disorders (*p* = 0.003). This condition corresponds to the action of NMDA receptor inhibitors, which intensify the appearance of psychosis, including increased positive and negative symptoms similar to those observed in schizophrenia.

**Figure 3:**
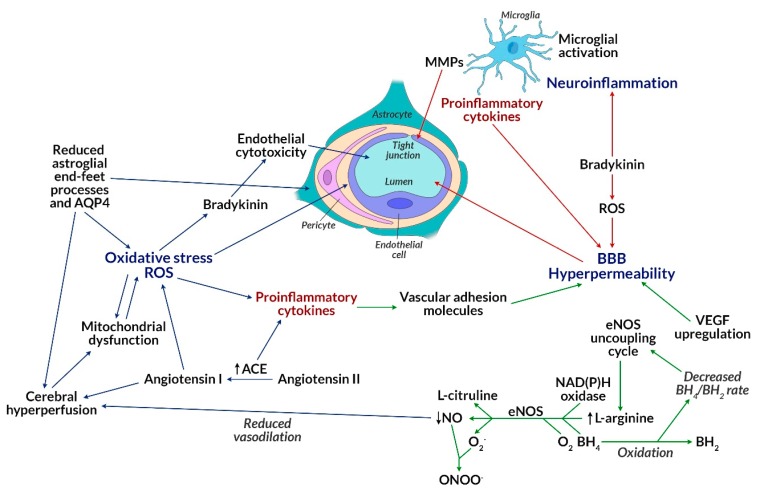
Impact of oxidative stress on blood-brain barrier (BBB) permeability. Excessive ROS, produced among others by mitochondria, may lead to oxidative stress, which may disrupt mitochondrial functions, as well as induce an increase in the level of proinflammatory cytokines. This consequently leads to an increase in the level of VCAM-1 and BBB permeability. Proinflammatory cytokines (formed after the activation of microglial cells) and ROS can also directly affect BBB permeability. The reduced BH_4_/BH_2_ ratio activates the eNOS uncoupling cycle, which increases L-arginine levels. Its increased level, as well as participation of NAD(P)H oxidase, BH_4_, O_2_, and eNOS leads to the formation of L-citrulline, NO, and superoxide radical (O_2_^•-^). NO reacts with O_2_^•-^, resulting in highly reactive, harmful peroxynitrite (ONOO^-^) forms, whereas the NO levels decrease. NO is responsible for vasodilatation of vessels. A decrease in its level causes a reduction in vasodilatation and, as a consequence, cerebral hyperperfusion. Cerebral hyperperfusion may be the result of reduced astrocyte-foot process and AQP4 levels. Their reduction can also directly lead to oxidative stress. The formation of angiotensin-I from angiotensin-II is catalyzed by ACE, which contributes to cerebral hyperperfusion, as well. Elevated ACE levels may promote the production of proinflammatory cytokines. Bradykinin can be both produced due to reactive oxygen species and lead to their formation. It exhibits a cytotoxic effect on endothelial cells and leads to inflammation of the nervous tissue. Stimulation of VEGF is also associated with an increased BBB permeability. Abbreviations: ACE: Angiotensin-converting enzyme; AQP4: Aquaporin 4; BBB: Blood-brain barrier; BH_4_/BH_2_: Tetrahydrobiopterin/dihydrobiopterin; eNOS: Nitric oxide synthase; MMP: Matrix metalloproteinase; NO: Nitric oxide; NAD(P)H: Nicotinamide adenine dinucleotide; O_2_^•-^: Superoxide radical; ONOO^-^: Peroxynitrite; ROS: Reactive oxygen species; VCAM-1: Vascular adhesion molecule; VEGF: Vascular endothelial growth factor.

Insulin resistance is associated with the inability of target tissues to increase glucose uptake in response to insulin [52]. Insulin resistance of brain tissues can reduce glucose levels in the brain by glucose-transporter-dependent pathways (among others, GLUT4), which together cause disturbed neurotransmission and disease progression [53]. Obese patients treated with atypical neuroleptics, in addition to insulin resistance, develop an additional phenomenon of lipotoxicity, leading to an increase in the level of free fatty acids (FFA) in the plasma, which is explained by their competition with glucose as oxidative substrates [54]. It can therefore be concluded that increased fat oxidation causes insulin resistance in obese people with schizophrenia. Elevated levels of cortisol, an insulin-dependent hormone associated with inhibition of lipolysis in adipose tissue, are also observed in those patients. As a result of secondary insulin resistance of target tissues (including adipose tissue), metabolic or hormonal changes occur, most commonly in visceral obesity through the pathway of multifactorial inhibition of insulin binding to the GLUT4 receptor located in adipocyte cell membranes [55]. This, in turn, induces further changes associated with the development of metabolic syndrome and type 2 diabetes observed during treatment of psychiatric patients with second-generation neuroleptics (e.g., olanzapine, clozapine, risperidone) [56,57]. In the absence of insulin or in case of insulin resistance of target tissues, as a result of triggering the mTOR pathway, the maturation and the development of dendritic branches is also impaired together with the proper functioning of neurons. Mammalian target of rapamycin (mTOR) is an integrated multi-protein serine-threonine kinase complex, existing in two functional forms of mTORC1 and mTORC2, which can be activated, among others by growth factors (insulin, insulin-like growth factor IGF-1), stress (DNA damage, decrease in blood glucose, and oxygen), amino acids such as leucine (Leu) and arginine (Arg), as well as high ATP levels [58,59,60,61]. Activation of each form of the complex is closely related to stimulation of the PI3K/PTEN/AKT/mTOR tyrosine kinase pathway, which hence linked to synthesis, autophagy, lipogenesis, ketogenesis, mitochondrial function, glucose uptake, as well as insulin signaling via the transmembrane receptor tyrosine kinase (RTK) [62,63]. The mTOR pathway controlling neurogenesis, synaptogenesis, cell proliferation, autophagy, and apoptosis, becomes a very important signaling pathway, whose disorder leads to serious consequences and is considered an important etiological factor of schizophrenia. Studies on autophagy-related gene mutations (ATG) in animals are largely associated with inability to survive [64]. This means that the observed changes in the mTOR pathway could be the single cause of nerve cell death, which means that their lifetime strictly depends on the above processes, such as autophagy. The essence of mTOR action is associated with the control of autophagy, which under physiological conditions prevents the accumulation of misfolded proteins (e.g., pentosidine) and removes abnormal organelles, including mitochondria, endoplasmic reticulum, ribosomes, and even synaptic vesicles (e.g., NR2A), which play a key role in the conduction of nerve impulses [65]. The mTOR-dependent autophagy disorder seen in schizophrenic patients leads to the formation of protein aggregates, which consequently promotes toxicity and cell death [66], as well as to altered synaptic conductivity and mitochondrial dysfunction (e.g., 22q11.2 deletion syndrome). mTOR may also cause neuronal insulin resistance, glucose deficiency (because insulin and amino acids directly affecting mTOR are the main regulators of ATG), and consequently reduced glucose metabolism, abnormal ATP production, as well as dysfunction and/or death of the cells. This pathway is involved in the translocation of GLUT1 receptors, which are then involved in glucose uptake, whose lowered levels indicate a disturbance in the mTOR pathway [67]. Adipose tissue taking part in the regulation of energy metabolism, glucose homeostasis, and insulin sensitivity, becomes insulin resistant if it contains an impaired mTOR pathway [68,69]. Signal pathways of 3-phosphatidylinositol kinase, inositol phosphatase (PI3K/PTEN/AKT/mTOR), protein kinase or mitogen activated kinases (MAPK/ERK) mediated by mTOR can be a mechanism explaining the formation of cardiometabolic complications of schizophrenia [70]. An animal model showing metabolic syndrome characteristics, where mTOR pathway inhibitors were administered, was analyzed. As a result, a reduction in oxidative stress (decrease in superoxide production and NADPH oxidase activity) and inflammatory response (reduction of macrophage influx), weight loss, reduction of adipose tissue hypertrophy, and hypertension were observed, which was beneficial to the cardiovascular system and reduced the risk of cardiometabolic complications [71]. Thus, cerebral insulin resistance in schizophrenia may induce reduced signal transduction for gamma-aminobutyric acid (GABA), *N*-methyl-d-aspartic acid (NMDA), dopamine-D2 receptors, and reduced levels of brain-derived neurotrophic factor (BDNF). KIF3B protein is involved in the transport of NR2A vesicles to *N*-methyl-d-aspartate receptors (NMDAR), which is crucial for neuronal plasticity and synapse formation. KIF3B plays an important role in the electrophysiological response of NMDAR and hence synaptic plasticity, which is one of the pathogenetic concepts of schizophrenia [72]. Although the presented studies concern the animal model, they are of great importance in patients with schizophrenia who were identified with KIF3B mutations. This confirms the abovementioned research and supports the pathogenic concept of schizophrenia. Neurexin, a presynaptic protein responsible for the proper joining of neurons into synapses, whose abnormal expression contributes to the loss of synapses and impaired signal transduction in the brain, is also directly related to the plasticity of neurons [73]. Together, this contributes to the inflammation of the local brain tissue, which is driven by local oxidative stress and occurs in the absence of energy substrates needed by the brain (Figure 3) [74].

One of the etiological concepts of schizophrenia relates to the immune system dysfunction, which mainly involves cytokines produced by T lymphocytes [75]. As is known, cytokines circulating in peripheral blood can interact with the brain through several mechanisms: Binding to specific transporters [76], stimulating the vagus nerve, binding to the receptors in peripheral organs, or through a changes in blood-brain barrier permeability with a S100B protein marker of damage [77]. Cytokines can also be produced by microglia cells, astrocytes, endothelial cells, and neurons (Figure 2 and Figure 3) [78]. An abnormal level of circulating cytokines is the cause of inflammation arising from the innate immune response and initiated by Toll-like receptors (TLR4) in the course of schizophrenia [79]. Changes in TLR4 levels affect the change in neuroplasticity, learning ability, and memory [80,81,82]. Currently conducted studies on the level of cytokines before and during the development of the disease aim to outline the therapeutic implications of the distortion of their levels and the usefulness of monitoring their concentration in schizophrenia [83].

Another pathogenic concept of schizophrenia emphasizes the role of reduced expression of mGlu2 and mGlu3 metabotropic glutamate receptors found in neuronal membranes. Their activation (by administering selective mGlu2/3 receptor agonists, e.g., LY379268, clozapine) causes antipsychotic effects manifested both in the form of reducing psychotic behavior and reversing existing molecular changes (by reducing the expression of DNMT1 and TET1). The antipsychotic effect is most likely due to DNA demethylation caused by growth-inhibiting and DNA-damaging Gadd45-β proteins [84,85].

An increase of mitochondrial function, as well as intensified cycles of citric acid and oxidative phosphorylation, are observed in patients with schizophrenia [86]. Velásquez et al. detected mitochondrial proteins that differ in their expression from normal levels. CLTC and PPIase A (proteins associated with endocytosis and protein folding) showed the reduced expression. An elevated level of eleven proteins associated with the citric acid cycle, electron transport pathway, and participating in the construction of the mitochondrial membrane has been detected [3], confirming the hypothesis of mitochondrial dysfunction in the etiopathogenesis of schizophrenia. It was discovered that neurons are deficient in generating bioenergetics substrates from glucose via the glycolytic pathways, which could result in TCA cycle abnormalities and impaired OXPHOS [87]. Increased glucose catabolism confirms the increase in plasma concentration of glycolytic enzymes, lactate, pyruvic acid, insulin, and GLUT1 [88]. The ratio of lactate and pyruvic acid concentrations is significantly reduced suggesting the predominance of pyruvate formation [88]. A different level of lactates can be observed in the cerebrospinal fluid (CSF), where its increase indicates an intensified extra-mitochondrial anaerobic glucose metabolism [89]. Imaging techniques and metabolomics assessing mitochondrial functions have allowed the determination of metabolites dependent on oxidative stress or inflammation to identify pathological changes in the brains of patients with varying degrees of schizophrenia [90,91,92,93]. Analysis of mitochondrial membrane redox potential (via phosphorous magnetic resonance spectroscopy, P-MRS) of brain tissue and peripheral metabolomics (analysis of blood or fecal metabolites) of patients with schizophrenia revealed that oxidative modifications of key glycolytic enzymes appear in the affected areas of the brain (e.g., pyruvate dehydrogenase, fructose bisphosphate, 3-phosphoglyceraldehyde dehydrogenase, phosphoglycerate kinase-1, phosphoglycerate mutase) [94]. Reduced levels of pyruvate dehydrogenase results in increased lactate and cellular acidosis and is closely associated with reduced levels of ATP production in the brain [95], which then leads to reduced glucose metabolism. Lipid peroxidation observed in schizophrenic patients causes changes in neuronal membranes, which consequently interferes with their function and affects signal transduction through changes in neurotransmitters and neuronal receptors located in postsynaptic membranes [96,97]. Oxidative damage also affects mitochondrial DNA [98], hence it can also contribute to impaired energy production, which is associated with free oxygen radicals, including superoxide anion radical O_2_^.-^ and hydrogen peroxide H_2_O_2_ [99]. Thus, both mitochondrial disorders and insulin resistance are closely related, because the oxidative stress produced in mitochondria interferes with the translocation of insulin-dependent GLUT4 transporters by accelerating the development of insulin resistance [100].

The metabolic syndrome developed during pharmacotherapy in patients with FEP or schizophrenia is a key reason for the insulin resistance of brain tissues, which is an important factor of cardiometabolic risk. Given the very large increase of worldwide metabolic syndrome and type 2 diabetes prevalence in connection with aging, schizophrenia is a serious and growing problem [101,102]. This review summarizes the role of oxidative damage and impaired glucose metabolism as key factors affecting metabolic complications in schizophrenia and highlights the role of a broader approach to schizophrenia by suggesting new therapeutic options.

The disease occurs long before the first clinical symptoms appear. The prodromal period lasts from several months to several years. Currently reported problems of the efficacy of schizophrenia pharmacotherapy may be associated with the late contact with medical personnel. The time between the onset of FEP and the start of pharmacotherapy varies between 22 and 150 weeks [103]. In addition, the diagnosis of neuropsychiatric disorders is largely based on subjective cognitive assessment. Due to the lack of commonly accepted central and peripheral biomarkers for the diagnosis of mental diseases, there is a need to search for them according to the newly emerging pathogenetic concepts of schizophrenia in order to objectify the diagnosis, which will have a direct impact on more effective therapy and less social burden.

## 2. Glycation and Oxidative Damage in Schizophrenia

During the reaction of reduction, carbohydrates react with the lysine amino groups of protein side chains to form advanced glycation end-products (AGEs). The creation of AGEs includes not only the direct reaction of amino groups of proteins with sugars, but also oxidative damage to proteins as a result of glycoxidation [104], or the formation of diacarbonyl products of the above reactions, i.e., methylglyoxal in the brain [105,106], or pentosidine as a peripheral biomarker of increased carbonyl stress [107].

Advanced glycation end-products (AGE) are proteins or lipids with sugar residues attached as a result of exposure to sugars in the glycation process with the participation of RAGE receptors (receptor for advanced glycation end-products) [108]. RAGE receptors are associated with the cell membrane of most body cells and can combine with various ligands, including AGE, amphoterin, β-amyloid, S100/calgranulin [108,109,110,111,112], which results in the activation of proinflammatory pathways that play an important role in the etiopathogenesis of schizophrenia. esRAGE subtype (endogenous secretory RAGE) is an unbound form of the RAGE receptor that protects cells from the harmful effects of AGE in the mechanism of blocking RAGE activation [113]. Reduced levels of esRAGE are observed in schizophrenic patients, which consequently causes increased exposure to carbonyl stress [114].

Glycoxidation is one of the key reactions of increased carbonyl stress both in the local brain tissue and in the peripheral blood of schizophrenic patients [114], partly because the reactive compounds of carbonyl stress form irreversible dysfunctional carbonylated multimer CRMP2 (collapsing response mediator protein 2) [115], which has a typical AGE structure and binds to RAGE in both neurons and endothelial cells. The binding of AGE and CRMP2 to RAGE generates further oxidative stress, which is a direct cause of inhibition of neurite growth, neuronal polarity, and transport of vesicles dependent on motor proteins, including kinesin superfamily proteins (KIF), regulating structural processes that affect synaptic transmission, and controlling the dynamics of microtubules in cells and developing brain [116,117,118].

Pentosidine classified as AGE has become an important peripheral marker indicating accumulation of glycation products in the body due to ease its determination [119]. Patients with schizophrenia show elevated levels of pentosidine with a simultaneous decrease in pyridoxal (active form of vitamin B6), which is involved in the detoxification of reactive carbonyl compounds (RCCs) and thus counteracts carbonyl stress [120]. Another compound that counteracts biological stress is betaine, whose levels are reduced in the course of schizophrenia, resulting in an increase in the concentration of carbonylated compounds, e.g., Nε- carboxyethyl-lysine (CEL) in the cerebrospinal fluid [121]. In the course of schizophrenia, reduced levels of glyoxalase 1 (GLO1) are also observed. This enzyme is responsible for the detoxification of methylglyoxal (MG) by its enzymatic conversion to less harmful compounds, e.g., D-lactic acid [122]. Studies in an animal model have shown a reduced GLO1 level in schizophrenia, resulting in an accumulation of methylglyoxal and thus, modification of behavior, e.g., increased anxiety and depressive states [123].

Glycation causes a number of undesirable effects in the body. It can slow down the transformation of proteins, which occurs through conversion into the structure of β-harmonica and fibrils, keeping them longer in toxic oligomeric forms [124]. Elevated levels of peripheral immune cells, such as macrophages and monocytes, are observed in schizophrenia [125]. These cells may contribute to the disturbance of both cholesterol transport from the blood and inflammatory response in the presence of AGE glycation products, which in turn may cause deformation of blood vessels and atherosclerosis [126]. Vascular disorders associated with protein glycation and the formation of toxic oligomeric forms are the cause of cognitive decline in schizophrenia [127].

## 3. Oxidative, Nitrosative, and Sulfuric Stress in Schizophrenia

Oxidative and nitrosative damage affects macromolecules (i.e., proteins, lipids, or nucleic acids) during oxidative and nitrosative stress. It occurs when there is an imbalance between the production of reactive oxygen species (ROS) and reactive nitrogen species (RNS) and antioxidants. Of importance are, inter alia, endogenous (i.e., superoxide dismutase SOD, catalase, peroxidase, glutathione, thiols, uric acid, bilirubin) and exogenous antioxidants (i.e., vitamins A (retinol), C (ascorbate) and E (α-tocopherol), carotenoids, lutein, ubiquitin, glutathione, polyphenols). Oxidative (OxS) or nitrosative (NxS) stress is very often associated with the pathogenesis of many diseases, including schizophrenia [128,129,130,131,132]. It may determine the further course of the disease [133] with progression or remission of symptoms [134]. The term ‘reactive’ is variable, which is due to the fact that some ROS and RNS are themselves highly reactive, e.g., hydroxyl radical (OH^•^) or nitric oxide (NO), while others become reactive only during reactions with other radicals, e.g., hydrogen peroxide (H_2_O_2_), nitric oxide radical (NO^•^), superoxide anion radical (O_2_^•−^) (Table 1).

The strongest and most aggressive of the reactive radicals in most biological systems is hydroxyl radical (OH^•^), whose standard potential value is equal to +1.902 ± 0.017 V [135]. This radical reacts with most organic and inorganic compounds including DNA (all purine and pyrimidine bases and deoxyribose chain), proteins, lipids, amino acids, sugars, and metals [3,7]. The effects of the hydroxyl radical are associated with increased lipid peroxidation and accumulation of its toxic products in patients with schizophrenia, which is strongly marked in the blood of patients [136]. That can bring a better understanding of important biochemical processes locally occurring in the body of these patients. The effects of lipid peroxidation in patients with schizophrenia also concern vasculitis and fibrosis [137]. Polyunsaturated fatty acids present in myelin sheaths are particularly sensitive to lipid peroxidation, becoming a direct cause of demyelination of axons in brain tissue [3]. 

Another self-reactive radical is superoxide radical O_2_^•−^, which acts as a mild oxidant in aqueous solutions, oxidizing ascorbic acid and thiols. Superoxide radical is a strong reducing agent for iron found in complexes, e.g., conjugated with cytochrome C or iron from EDTA [3,82]. Irreversible oxidation of iron-sulfur centers leads to inactivation of the abovementioned complex [7]. As a result of the reaction of O_2_^•−^ with highly reactive nitric oxide (NO^•^), harmful nitrogen forms are formed, e.g., peroxynitrites (ONOO^-^) [3], thanks to which the bioavailability of nitric oxide is reduced, and thus, the proper blood supply to the vessels is disturbed [1]. This is confirmed by our previous observations related to the reduced production of peripheral NO_2_ˉ in patients with the first episode of psychosis, compared to patients with normotension. The research conducted by Śmierciak et al. confirmed the relationship between cardiovascular risk associated with inflammation (CRP), lipid profile, and NO_2_^−^ production in patients with FEP. This proves that peripheral endothelial dysfunction is associated with reduced NO_2_^−^ production. Therefore, modification of endothelial function by increasing NO_2_^−^ levels appears as a new therapeutic strategy for patients with FEP [138].

Hydroperoxide HOO^•^, ozone (O_3_) [82], and singlet oxygen (½ O_2_) [1,2] are responsible for peroxidation of lipids, proteins, and nucleic acids, and are more potent oxidant/reducing agents than superoxide radical. They also have the ability to cross cell membranes into the extracellular space [2] and to mediate cell signaling [1].

Independently reactive radicals include reactive nitrogen species (RNS) and nitric oxide (II)/(IV) (NO^•^), which is a gaseous signal molecule, responsible for vasodilatation and neurotransmission. Intermediate products formed with the participation of nitric oxide may lead to necrosis of liver cells, inhibition of mitochondrial function and depletion of pyrimidines, which may result in disruption of the DNA double helix [3]. Nitric oxide has an effect on ATP production by affecting the electron transport of the respiratory chain. In the brain, nitric oxide and nitrogen dioxide affect the complexes I, II, IV, and V of the respiratory chain [78]. The basic biochemical reactions involving nitric oxide are: S-nitrosylation of thiols, and nitrosylation of transition metal ions [12,79,80]. The structure of hemoglobin can be changed by direct attachment of NO to the heme in the nitrosylation or *S*-nirosylation of thiols, when *S*-nitrosothiol is formed. Forty five S-nitrosylated proteins were identified in different regions of the brain (hippocampus, black matter, cerebral cortex) of Alzheimer’s disease patients, 15 of which were involved in metabolism (including lactate dehydrogenase) [78]. Glutathione (GSH) can also undergo S-nitrosylation. In postmortem studies, glutathione reductase levels correlated with the age of schizophrenic patients. In the absence of antipsychotic treatment, GSH levels may be reduced [79]. NO and peroxynitrite have a cytotoxic effect on oligodendrocytes [5]. Peroxynitrite (ONOO^-^) is another strong oxidant to many biologically active molecules. NO and ONOO^-^ can react with each other. As a result of this reaction, hydrogen peroxide is formed, which inhibits mitochondrial respiration, Na^+^/K^+^ pump function, and phosphorylation of kinases [3]. Peroxynitrite may cause nitration (e.g., of protein tyrosine residues) and hydroxylation. In blood vessels, it reacts with carbon dioxide (CO_2_). In cells, it oxidizes thiols [81]. The reaction of peroxynitrite with transition metals may form a hydroxyl radical [3]. Peroxyacid (ONOOH) is a protonated form of peroxynitrite. It is a strong oxidant produced by activated macrophages, thus showing a positive effect. It may cause oxidation of thiols, ascorbate, and lipids [81]. Peroxyacid can destroy sulfhydryl groups and cause oxidative destruction of biomolecules, acting similarly to the hydroxyl radical. It has cytotoxic effects on cells and mitochondria, causes DNA breaks, protein oxidation, nitration of aromatic amino acids, resulting in 3-nitrotyrosine [3].

Among radicals that are not reactive independently but reactive with other radicals, both ROS and RNS are distinguished. Such ROS include, among others, hydrogen peroxide H_2_O_2_, which is a weak oxidant and a weak reducer [82]. As a result of reaction with metal ions (all group D metals except zinc, e.g., iron and copper), it creates highly reactive radicals including HO^•^ in Fenton reaction [7,83], whose role in free radical damage to schizophrenic patients was mentioned above. As a result of the direct action of hydrogen peroxide, the structures of heme proteins are modified with the release of iron. Hydrogen peroxide also causes inactivation of enzymes and oxidation of DNA, lipids, -SH groups, and keto acids [3]. Hypochlorous acid (HOCl) has a high oxidative capacity. Its action may lead to oxidative halogenation of biomolecules such as lipids, proteoglycans, amino acids, as well as other membrane components and intracellular elements [3]. Organic peroxides (ROOH) are another group of independently nonreactive ROS. They are formed in the reaction of HO_2_ with RO_2_, mainly in the oxidation of alkanes and alkenes [85]. Organic peroxides are produced by, among others, α- and β-pyrene, isoprene, limonene, as a result of the decomposition of organic hydroperoxides in the presence of Fe^2+^ in the Fenton reaction (OH· is also formed then).

Nitroxyl (HNO) is an example of independently reactive RNS. It can cause direct oxidation of thiols, which distinguishes it from NO. In the cardiovascular system, it activates cardiac thiol-containing proteins, e.g., sarcoplasmic ryanodine receptors (RyR2). It causes vasorelaxation by activating soluble guanyl cyclase (sGC) and further increases in cGMP. It can activate ATP-sensitive and voltage-gated potassium channels and affect the release of calcitonin gene-related peptide. It causes reduction of vascular O_2_^•-^ production. It also has vasoprotective and cardioprotective effects [84]. As a result of the reaction of NO with NO_2_, a nitrosyl cation (NO^+^) [88] and a nitrosyl anion (NO) [81] are formed. The cation is formed through oxidation, and the anion through reduction. Nitrosyl cation is responsible for the post-translational modification of thiol-containing proteins, i.e., S-nitrosation of their thiol groups [88], which can lead to homolysis of S-N bonds. It is accompanied by the release of NO, formation of S-S bonds, and conformational changes affecting the cell. Nitrosyl cation probably can also cause the formation of S-nitrosamines. Nitrosyl cation may be present in complexes or transported, e.g., by S-nitrosoglutathione. NO^+^ donors have been shown to effectively affect Swiss 3T3 fibroblast and PC12 neuronal cell apoptosis [81]. Some studies suggest that nitrosyl anion may be formed directly by nitric oxide synthase (NOS). Nitrosyl anion reacts with oxygen resulting in the formation of peroxynitrite (ONOO^-^), which occurs during the oxidation of hydroxylamine in an alkaline environment. Attachment of NO to heme Fe^2+^ produces NO-nitrosyl heme, from which NO^-^ can be released. It is believed that nitrosyl anion may be involved in the degradation of S-nitrosothiols in the presence of thiols. Nitrosyl anion may be responsible for reactions typical of endothelial-derived relaxing factor (EDRF) and cause double-stranded DNA breaks [81].

Similar to ROS and RNS, reactive sulfur forms (RSS) perform important biological functions such as cell signaling, redox homeostasis, and metabolism regulation [106]. RSS are formed as a result of one-electron oxidation of hydrogen sulfide (H_2_S). As a result of a number of reactions, sulfhydryl radical (HS^•^), thiyl radical (RS^•^), and persulfide radical anion (‘supersulfide’ (HS_2_^•−^) are formed [135,139,140]. Further oxidation of hydrogen sulfide to sulfane sulfur (S0; thiosulfoxide) produces sulfanes (including hydrogen disulfide H_2_S_2_, sulfenic acids RSOH, and elemental sulfur (S)) [139]. Sulfates can then react with another sulfane sulfur to form polysulfides. Organic RSS can also arise from thiol (RSH) oxidation [141].

Hydrogen sulfide (H_2_S) can be synthesized from cysteine, homocysteine, thiosulfates, and carbonyl sulfide as a result of many different reactions occurring in the cytoplasm and mitochondria [139]. It has a protective function against OxS and inflammation, as well as a signaling function. The signaling role of H_2_S is associated with post-translational oxidative modification of cysteine residues resulting in cysteine persulfides. H_2_S may reduce disulfide bonds causing disruption of enzymatic function. It can be released in an acidic environment (but not under physiological conditions) from the iron-sulfuric complex belonging to the respiratory chain. SOD oxidizes hydrogen sulfide to polysulphides, which can then react with subsequent sulphides to form long-chain polysulphides. SOD metabolizes sulfides to disulfan (H_2_S_2_). Polysulphides are also formed, among others, in neurons as a result of the transfer of sulfuric ion from 3-mercaptenopyruvate to cyanides, other thiols, which catalyzed by 3-mercaptopyruvate sulfurtransferase (MPST), e.g., H_2_S_2_ and trisulfan (H_2_S_3_) soluble in the brain [142]. In addition to the sources of sulfans and polysulphides mentioned above, they are also generated in oxidation of hydrogen sulfide by unbound or heme-bound transition metals, myeloperoxidase, catalase, superoxide dismutase, and other oxidases, as well as the result of H_2_S metabolism and cysteine catabolism. Polysulphides can play a protective role against thiols in conditions of oxidative stress. Disulfanes and polysulfides can be both oxidants and reducing agents, can be used as signaling molecules or as an energy source. Polysulfides can protect neurons from oxidative stress by activating Nrf2/Keap1 (nuclear factor erythroid-2-related factor-2/Kelch-like ECH-associated protein 1) pathway. Polysulfides have been shown to neutralize methyl glyoxal and inhibit its cytotoxicity [142].

Although H_2_S and polysulphides are necessary for proper functioning, recent studies have shown that their elevated levels can lead to the development of pathophysiological processes and brain disorders. MPST expression was elevated in the brains of people with schizophrenia. This suggests that patients subjected to sulfuric stress have stronger psychotic symptoms. Studies show that elevated H_2_S levels in schizophrenic patients already occur at an early stage of the disease development [89]. Blood and plasma tests of schizophrenic and FEP patients showed elevated homocysteine levels, which probably correlates with the severity of the symptoms. In turn, the level of hydrogen sulfide in the plasma of patients with schizophrenia was reduced and showed a negative correlation with the severity of the symptoms. Impaired hydrogen sulfide synthesis may impair the physiological effects of NO, which may worsen the symptoms of schizophrenia [142].

Intracellular cysteine hydropersulfide (CysSSH) is involved in 2-methylthio-modifications of t-RNA in mammals, and also affects insulin secretion [7].

Thiyl radicals can also react with OH^•^ causing the formation of very reactive sulfenic acids, which can, among others, form an intramolecular disulfide bond with cysteine and react with low-molecular thiols. Sulfenic acids are irreversibly oxidized to sulfinic acid (–SO_2_H) and sulfonic acid (SO_3_H) [10,143].

These processes result in an increase in oxidative, nitrosative, and sulfuric damage, which may include impaired glucose metabolism and the associated loss of ion gradients depending on functional potential disturbances and Ca^2+^ transport [144]. During oxidative stress, there is an intracellular increase in free Ca^2+^ levels, which are known to have unfavorable consequences, including depolarization of mitochondrial membranes, oxidative phosphorylation disorders, and overproduction of free radicals. Moreover, research in schizophrenic patients suggests that changes in mitochondrial function due to ROS occur before treatment. Hence, the mechanisms of action of psychiatric drugs are both direct and indirect, suggesting that free radical damage is an etiopathological factor in schizophrenia [91,145].

As a result of these changes, vital neurons of the frontal cortex, hippocampus, and other regions, which are physiologically responsible for normal synaptic plasticity and neurotransmitter release are turned off [24,25,26,27]. These neurons affect learning and memory, focus, cognitive functions, and are responsible for the organization of neurons in the finely-tuned circuits and motor functions during puberty [27,28].

In addition, DNA oxidative damage, e.g., 8-hydroxydeoxyguanosine, can interfere with gene transcription and affect promoter function, which can lead to impaired transcription of essential genes, i.e., suppressor genes, p53 gene, ras gene, and further mutations. In turn, RNA oxidative damage can damage protein translation, which leads to reduced expression of proteins and loss of their physiological functions (Table 2) [18,36,37].

Oxidative, nitrosative, or sulfuric damage occurs when specific ROS/RNS/RSS react with macromolecules, which can be identified by measuring specific reaction products that induce the abovementioned damage referred to in the literature as ‘oxidative, nitrosative and sulfurative biomarkers’ [128,146,147,148]. The most frequently appearing terms for commonly used biomarkers of oxidative damage to macromolecules, i.e., lipids, proteins, and nucleic acids are summarized in Table 2.

Oxidative, nitrosative, sulfur (SxS) stress, and inflammation disturb brain development [203]. The stress can affect neuronal plasticity, signal transduction, and induce apoptosis [129,204]. It is believed that OxS markers and inflammation may be important indicators of the course of schizophrenia and provide information on the disease progression and the effectiveness of treatment [128]. The brain of schizophrenic patients contains increased levels of oxidative/nitrosative/sulfuric damage products, i.e., 4-hydroxy-2-nonenal (HNE), 3-nitrotyrosine or homocysteine, associated with the trans-sulfur pathway [143]. Interestingly, the increase in HNE was associated with the accumulation of iron in the black matter, a region of the brain heavily affected by pathology [175], while an increase in 3-nitrotyrosine levels was seen in the prefrontal cortex of patients with psychosis [205]. In the case of antioxidative system indicators, there was a decrease in the activity of key antioxidative enzymes, i.e., glutathione peroxidase (GPx) or superoxide dismutase (SOD). The aforementioned intensification of lipid peroxidation expressed by increased levels of malondialdehyde (MDA) was identified in the blood of people with psychosis [206], although these changes did not correlate with executive functions (EF) in patients with chronic schizophrenia [206].

In relation to carbonyl stress in the course of schizophrenia, hyper-carbonylation of a collapsin response mediator protein (CRMP2) [115] was observed in the brain and cerebrospinal fluid of patients. Increased levels of carbonylated proteins in the cerebrospinal fluid of the studied patients support the theory of carbonyl stress [157]. Interestingly, increased levels of AGEs, which are also significantly associated with carbonyl stress in schizophrenia, were characteristic of people with treatment-resistant disease [207]. In contrast, esRAGE, which is one of the soluble forms of RAGE, is considered to be a key carbonyl stress suppressant by binding to AGEs. As indicated, lower levels of both esRAGE and sRAGE in the serum can be associated with the functional haplotype of people with schizophrenia in whom AGEs are the main cause of carbonyl stress [114].

The consequences of OxS/NxS/SxS in the brain may include disorders of glucose metabolism and thus disturb the ion gradient with impaired conductivity in the area of action potential and Ca^2+^ imbalance [157]. Oxidative stress increases intracellular levels of free Ca^2+^ which can lead to impaired energy supply. Energy in the form of ATP necessary for the proper functioning of neurons originating from oxidative phosphorylation from the inner mitochondrial membrane can be regulated, inter alia, by intracellular Ca^2+^ level [207,208]. Ca^2+^ stored in the endoplasmic reticulum (ER) is quickly and effectively transported to the mitochondria through mitochondria-associated ER membranes (MAM). MAM has a DISC1 (disrupted-in-schizophrenia 1) on its surface—a scaffold protein that is credited with affecting the function of intracellular organelles, i.e., mitochondria, centrosomes, endosomes, lysosomes, and affecting receptor degradation, as well as regulating intracellular transport [209] associated with cognitive and personality disorders [210]. DISC1 reacts with the type 1 inositol 1,4,5-triphosphate receptor (IP3R1) and inhibits its binding to the inositol triphosphate (IP3) ligand, affecting Ca^2+^ transfer through MAM between ER and mitochondria. DISC1 mutations cause oxidative/carbonyl stress during which hydrogen peroxide, superoxide anion, and C_2_-ceramide impair the transport of Ca^2+^ in mitochondria, leading to its penetration from ER through IP3Rs. The presented mechanism becomes the cause of depolarization of mitochondrial membranes, oxidative phosphorylation disorders, and overproduction of free radicals, resulting in the exclusion of vital neurons of the frontal cortex, hippocampus, etc., which are physiologically responsible for the normal synaptic plasticity and the release of neurotransmitters [211,212,213,214]. Thus, they affect learning and memory, focus, cognitive functions, and are responsible for the organization of neurons in the finely-tuned circuits and motor functions during puberty [214,215].

Studies show that there are other mechanisms of Ca^2+^ transport that affect mitochondrial function. Thus, mitochondrial ryanodine type 1 receptor (mRyR1) found in cardiac cells and neurons is better suited for Ca^2+^ transport between endoplasmic reticulum and mitochondrion and plays the key role in mitochondrial uptake of Ca^2+^ [216]. Increased levels of Ca^2+^ in the cytoplasm causes Ca^2+^ to accumulate in the mitochondrial matrix and consume the mitochondrial membrane potential that affects the mitochondrial ability to produce ATP [217] and regulate dehydrogenase activity in the citric acid (Krebs) cycle (glycerophosphate dehydrogenase, pyruvate dehydrogenase, isocitrate dehydrogenase, α-ketoglutarate dehydrogenase) [216,217]. Reversible phosphorylation of the Ca^2+^-mediated pyruvate dehydrogenase complex partially regulates the supply of reducing equivalents. Activation of the Krebs cycle increases the production of nicotinamide adenine dinucleotide (NADH), which causes the electrons of the complex I of the respiratory chain to move to complex IV [217]. Activation of these reaction chains leads to increased ATP synthesis [216]. These disorders are associated with working memory deficits and changes in the short-term hippocampus strengthening [218] and impairment of motor skills [219].

The respiratory chain I complex and the cytochrome c oxidase subunit are regulated by specific protein kinases and phosphatases [220,221]. Cyclic-adenosine-3′,5’-monophosphate-dependent kinase catalyzes the phosphorylation of the complex I subunits. When the Ca^2+^ level is low, phosphatases dephosphorylate and inactivate the complex I [217], whereas when the Ca^2+^ level is high, mitochondrial phosphatase dephosphorylates cytochrome c oxidase and inactivates its allosteric inhibition [222].

Uncoupling proteins catalyze the controlled leakage of protons from the mitochondria through the inner membrane. They may be associated with the regulation of Ca^2+^ uniport, which affects Ca^2+^ homeostasis and, hence, the ATP level. Uncoupling proteins 2, 4, and 5 are present in the central nervous system and are thought to protect neurons from excess Ca^2+^ and OxS. Uncoupling protein 4 regulates Ca^2+^ homeostasis and affects Ca^2+^ influx into the endoplasmic reticulum [217].

In addition, DNA oxidative damage by, e.g., 8-hydroxydeoxyguanosine, can interfere with gene transcription and affect promoter function, which can lead to impaired transcription of essential genes, i.e., suppressor p53 ras genes, and further mutations. In turn, RNA oxidative damage can impair protein translation, which leads to reduced expression of proteins and loss of their physiological functions (Table 2) [157,223,224].

Traditional OxS identification methods focus on molecular biomarkers that are determined by chemical analytical methods [128,225,226,227,228,229,230,231,232,233,234,235,236].

The antioxidant defense system is designed to protect against the harmful effects of free radicals. In the literature, one can find many studies devoted to particular antioxidants, whose activity or concentrations correlate with the course of free radical reactions [232,236]. Cooperation between different antioxidants results in a greater protective effect than would result from the sum of the antioxidant effects of each compound separately [130]. There are various terms in the literature regarding total antioxidant capacity (TAC), which examples are presented in Table 3.

The estimation of the total antioxidant capacity as a clinical exponent of OxS is associated with a different approach consisting in the determination of:

- Concentrations of single or all low molecular weight antioxidants in plasma, serum, saliva, and other biological materials [92,134];
- Activity of selected enzymes [92,249];
- OxS markers, e.g., 8-hydroxyguanosine or MDA as exponents of lipid peroxidation [92,249,250].

Total antioxidant capacity of plasma is the result of the action of low molecular weight antioxidants (e.g., α-tocopherol, ascorbic acid, β-carotene, glutathione, uric acid, bilirubin), proteins (e.g., ceruloplasmin, ferritin, albumin, transferrin), and enzyme systems [130,131,132]. OxS-induced decrease in TAC is eliminated by the increase of antioxidant enzyme activity and activation of nonenzymatic mechanisms. In the latter phase of OxS, a decrease in TAC is observed, which is caused by the exhaustion of antioxidant defense mechanisms [251].

The clinical significance of antioxidative potential determination is associated with the assessment of the increased risk of certain diseases and their complications (including schizophrenia) or the impact of treatment on compensatory elements associated with the antioxidant defense of the organism [92,129,240,252]. Another approach proposes to use a single general OxS marker, which is a moderately strong oxidant K_2_IrCl_6_(VI)—Ir^OX^, to obtain chemical data on OxS. This approach does not focus on the determination of a specific chemical compound, but detection of environmental characteristics that indicate disturbances between oxidative and reducing activity [128].

Interestingly, serum OxS determinations correlate with the occurrence and course of schizophrenia, which suggests that OxS in CSN occurs earlier which may be identified already in the early stages of the ongoing pathology. In addition, OxS data correlate with indicators of damaged BBB in schizophrenic patients, which combines the concept of oxidative stress and neuritis [128] with the pathogenesis of schizophrenia [253,254]. Morphological changes in the structure of capillaries and cells of the neurovascular unit (NVU) occur in the prefrontal and visual cortex of schizophrenic patients, along with vacuole destruction of endothelial cells, astrocyte-foot processes, and the reduction of baseline membrane thickness [254] (Figure 2 and Figure 3). Changes in tight junctions are observed in the brains of schizophrenic patients [253,255], which are reflected in the reduction of expression of 12 out of 21 genes associated with their formation compared to the control group [254]. It has been shown that tight junctions can be broken [253], which has been associated with the presence of large areas lacking claudin-5, whose expression is reduced in the prefrontal cortex of patients with schizophrenia [253,254,255]. Claudin-5 proteins are absent along the blood vessels of the brain of schizophrenic patients, which may be associated with the penetration of low-molecular blood elements, e.g., S100β, resulting in impaired brain homeostasis [253]. The calcium-binding S100β protein is mainly produced by astrocytes [253,256,257]. An increase in plasma S100β levels shows a positive correlation with the occurrence of negative symptoms of schizophrenia and cognitive disorders [254].

The relationship between neuroinflammation, oxidative stress, endothelial damage, and impaired mitochondrial metabolism due to the dysfunction of the blood-brain barrier in patients with schizophrenia is presented in Figure 3. Vascular disorders associated with hyper-permeability of the blood-brain barrier can impair many functions simultaneously: They can affect cognitive and behavioral functions associated with neuroinflammation in the microglial area, which leads to a deepening process of neurodegeneration in the CNS [258]. According to the conception of de la Monte [259], neurodegeneration may be called type III diabetes or CNS metabolic disease, where it comes to an increase in oxygen, nitrogen, and sulfuric free radicals (ROS, RNS, RSS) [260] due to activation of inflammatory pathways (and increased release of proinflammatory cytokines, e.g., IL-6, IL-1B, TNF-α, by glial cells and astrocytes) [261]. It causes mitochondrial damage and activation of a number of kinase pathways, e.g., MAP, JNK, and p38MAPK, which may also result in inhibition of insulin signaling [262]. Disorders of glucose metabolism in the CNS increase the production of ketone bodies and the activation of additional pathways mediated by free radicals, including NF-kB [7]. According to the literature data, insulin axis impairment and hypometabolism in the CNS cause loss of neurons, astrocytosis, and microgliosis mainly within amygdala and septum [263], axonal damage and demyelination of the hippocampus and fornix [264]. McNay et al. indicate that insulin signaling and abnormal glucose transport significantly affect cognitive processes and memory functions regulated by the hippocampus [265]. This is due to the incorrect activation of the insulin-sensitive GLUT4 transporter on the periphery and GLUT1–3 in the brain tissue [10]. Abnormal glucose metabolism can also affect the integrity of white and gray matter due to the effect of hyperglycemia on the dynamics and function of mitochondria, which leads to the accumulation of ROS and impaired axonal transport [266], mainly within the thalamus, parietal lobe, and hippocampus [267].

Progression of schizophrenia is increasingly associated with the treatment-resistant (TRS) form of the disease. The severity of symptoms is the result of sulfuric stress and is positively correlated with high levels of homocysteine and the failure of transsulfuration process, which metabolizes homocysteine in the l-cysteine pathway [143,268]. l-cysteine deficiency and lack of response from the transsulfuration pathway (low activity of cystathionine β-synthase and cystathionine γ-lyase) become the cause of the recorded low levels of glutathione and glutathione pathway enzymes (i.e., GPx) in the brain and blood of patients with schizophrenia [269]. As a consequence, the level of hydrogen sulfide (H_2_S) decreases and the oxidative phosphorylation (OXPHOS) pathway containing a number of proteins that have iron and sulfur in their structure is impaired. During sulfuric stress, the synthesis of these iron and sulfur-containing proteins is deregulated, which directly strikes OXPHOS, which becomes inefficient in schizophrenia. Consequently, the molybdenum cofactor (MoCo), which requires sulfur (from L-cysteine), affects the dependent sulfite oxidase (SUOX), which detoxifies sulfites from sulfur-containing amino acids. If the SUOX is low, sulphite toxicity, which is a component of oxidative-nitrosative-sulfuric stress, may occur, which may result in a decrease in the synthesis of selenoproteins, i.e., GPx, observed in schizophrenia and its first symptoms [270]. Sulfuric stress increases damage to macromolecules, i.e., DNA or proteins (in the carbonylation process), leading to methylation of genes and histones, affecting epigenetic changes in TRS.

Although H_2_S and polysulfides are necessary for proper functioning, recent studies have shown that their elevated levels can lead to the development of pathophysiological processes and brain disorders. MPST expression was elevated in the brains of people with schizophrenia. This suggests that patients with this disease subjected to sulfuric stress have stronger psychotic symptoms. Studies show that increased levels of H_2_S in schizophrenic patients occur at an early stage of the disease development [89].

The presented pathogenetic mechanisms related to oxidative, nitrosative, or sulfuric stress, impaired glucose metabolism, and mitochondrial dysfunction may constitute a therapeutic target for existing antipsychotic drugs and alternative therapies for TRS. It could be directed at the disorders of oxidative-antioxidative regulation, mitochondrial metabolism, as well as regulation of other pathways, resulting from glucose metabolism dysregulation.

## 4. Conclusions

Observations in this manuscript, as well as our own research (papers are being prepared), is strengthening the theory of impaired glucose metabolism at an early stage of schizophrenia. Our paper attempts to bring the recipients closer to a multidisciplinary approach to schizophrenia, as a disease involving the complexity of the brain pathology process, in correlation with peripheral changes.

This article aims to draw attention of the public, mainly physicians dealing with the younger generation, to expand the panel of diagnostic tests to facilitate the assessment of impaired glucose metabolism as a factor determining the state of the brain and the progression of mental illness over time. Research in this area can be helpful in reduction of stigma associated with the disease, and can be useful in the process of monitoring the treatment of affected individuals.

The presented manuscript points out scientific evidence to the role of oxidative, nitrosative, sulfuric antioxidative damage, which is a key element of pathomechanism and progression of schizophrenia and its symptoms. Glucose metabolism, which is the main source of energy for the brain, is impaired in schizophrenia and the first symptoms of psychosis. These changes are largely due to impaired oxidation of key glycolytic proteins or energy production in the form of ATP. Smaller energy supplies to the ‘necessitous’ brain cause a number of changes including the exclusion of vital functions, such as impaired functional potential or increased levels of intracellular Ca^2+^, altering the basic functions of neurons.

Reactive forms of oxygen, nitrogen, or sulfur are the cause of oxidative, nitrosative, and sulfuric damage inducing lipid peroxidation, protein carbonylation, or glycation. This creates reactive molecules that critically affect the proteins of the mitochondrial metabolism in the brain, changing its functions and promoting the symptoms of schizophrenia.

Presynaptic membranes are particularly sensitive to oxidative stress, leading to impairment of cognitive functions and motivation, and ultimately to impairment of perception of reality. Affected neurons die, leading to the clinical manifestation of schizophrenia. As a neuropathology, it may be preceded by a prodrome or may be resistant to treatment, hence understanding the pathomechanism of the disease and increased efforts to better understand the biochemical, genetic background, or imaging techniques assessing anatomical or functional changes in the brain, seems necessary.

Given the increasing percentage of people with diabetes or cardiovascular disease in the group of people with schizophrenia along with an aging population, impaired glucose metabolism or insulin resistance seem to be more common in the general population. They require targeted therapies aimed at impaired glucose metabolism and reducing oxidative, nitrosative, and sulfuric stress, and thus protecting the brain from threatening neuropathological changes disabling its basic functioning.

In this work, an attempt was made to present the main pathogenic pathways of schizophrenia caused by an oxidative-antioxidant imbalance that impairs glucose metabolism, leading to the weakened neural conduction and neuronal death.

Considering the role of individual brain cells, i.e., glial cells associated with bioenergetics of brain processes, glucose metabolism dysfunctions, and accelerated aging of neurons in schizophrenia, current research should focus on the mechanisms highlighted in this paper. That can significantly contribute to understanding the consequences of this disease already at the stage of its early symptoms. This in turn can lead to the development of effective therapeutic strategies for those struggling with this severe disease.

In our further research, we will present the results of events regarding the sequence of changes described in this manuscript, which should also be taken into account trying to determine the pathomechanism of the disease and understand the development of treatment-resistant forms, as well as a strategy for more effective therapy directed at oxidative stress, impaired glucose metabolism, and neuronal death.

Available imaging techniques in combination with effective biomarkers of disease progression stand open to new possibilities of testing schizophrenic patients and open potential new ways of treatment, which hopefully will arise for a new generation of patients suffering from schizophrenia.

## Figures and Tables

**Table 1:** Selected biologically important reactive oxygen species (ROS), reactive nitrogen species (RNS), reactive sulfur forms (RSS).

	ROS	RNS	RSS
Reactive independently	Hydroxyl radical (OH^•^)	Nitric oxide (II)/(IV) – nitric oxide and nitrogen dioxide (NO^•^ and NOO)	Sulfhydryl radical (HS•)
			Thiyl radical (RS•)
	Perhydroxyl radical, the protonated form of superoxide radical (HOO·)	Peroxynitrite (ONOO-)	Persulfide radical anion, ‘supersulfide’ (HS_2_^•−^)
	Superoxide radical (O_2_^•−^)	Protonated form of peroxynitrite (peroxyacid) (ONOOH)	Sulfenic acids (RSOH), e.g., sulfinic acid (–SO_2_H) and sulfonic acid (–SO_3_H)
	Ozone (O_3_)		Sulfans, np.: Hydrogen disulfide (H_2_S_2_), trisulfan (H_2_S_3_)
	Singlet oxygen (½ O_2_)		Polysulfides (H_2_S_n_)
Not reactive independently, reactive with other radicals	Hydrogen peroxide (H_2_O_2_)	Nitroxyl (HNO)	Cysteine hydropersulfide (CysSSH)
	Hypochlorous acid (HOCl)	Nitrosyl cation (NO^+^)	Thiols (RSH)
	Organic peroxides (ROOH)	Nitrosyl anion (NO^-^)	Hydrogen sulfide (H_2_S)

**Table 2:** Selected peripheral and brain biomarkers of oxidative damage. Biologically important damage to proteins, lipids, and nucleic acids caused by oxidative stress.

ROS Target	Directly Measured Product of Oxidative Damage	Mechanisms	Effects
Proteins	3-nitrotyrosine	Produced during radical–radical reaction between: Superoxide radical (O_2_^•-^) with nitric oxide (NO^•^) to form peroxynitrite (ONOO^−^), which leads to nitration of Tyr residues in proteins; between the tyrosyl radical and nitrogen dioxide (^•^NO_2_). Moreover, occurs during reactions catalyzed by peroxidases, including myeloperoxidase (MPO) or eosinophilic peroxidase. 3-Nitrotyrosine is also formed in a mechanism catalyzed by modified superoxide dismutase (Cu, Zn-SOD), which has a greater ability to catalyze the nitration reaction of tyrosine residues caused by ONOO^−^. This occurs in motor neurons [149].	Affects the structure and function of proteins in vitro and in vivo; present in inflammation associated with vascular endothelial dysfunction and cardiovascular complications in schizophrenia [150]. Correlates with the severity of atherosclerotic lesions; serves as an independent indicator of the risk of coronary artery disease in patients with FEP or schizophrenia. Biomarker, whose level depends on the pharmacotherapy, e.g., clozapine induces oxidative and nitrosative stress (in the caspase-3, NF-κB p65 or Nrf2 mechanism) depending on the dose in the cardiovascular system [151,152,153].
	Protein carbonyls	Carbonylated proteins are formed in the course of the following mechanisms: 1. The formation of aldehydes due to the cleavage of the peptide chain resulting in the formation of free radicals that convert into aldehydes; 2. Formation of oxidized amino acid side chains (oxo-histidine); 3. Combining proteins with lipid peroxidation products; 4. Creating advanced glycation end-products (AGEs).	Protein carbonyls increase: Carbonyl stress; production of conformationally altered polypeptide chains, which contributes to cellular dysfunction; excessive aggregation of proteins by promoting unfolding and formation of noncovalent, as well as covalent bonds between proteins; increased toxicity; may lead to apoptotic cell death [154,155].
	Dityrosine	Can be formed as a result of metal-catalyzed bond formation between two tyrosine residues. The reaction proceeds to form a tyrosine radical that isomerizes the entire aromatic ring. As a result of the combination of two radicals in the ortho–ortho position, an unsaturated bis-ketone is formed, which leads to the formation of dityrosine [156]. The change is irreversible [157].	Affects the redox state disorder; elevates the level of inflammatory factors, causing oxidative damage to the hippocampus; contributes to the deterioration of learning and memory skills [158]. It may contribute to the amino acid metabolism disorder and inhibit energy production [159].
	Methionine sulfoxide	Hydrogen peroxide reacts with methionine residues at pH = 5 to form methionine sulfoxide. Under these conditions, cysteine residues are resistant to methylation. Cysteine thiol group must first be ionized in order to be oxidized. At acidic pH, methionine is oxidized by dimethyl sulfoxide to methionine sulfoxide. At neutral or alkaline pH it is oxidized under the influence of hypochlorous acid, oxygen, ozone, peroxynitrite, superoxide radical [160]. Under the influence of further oxidation, methionine sulfoxide can be converted to methionine sulfone [157].	Methionine sulfoxide reductase is present in many organs, including the brain. It has a protective function against the effects of oxidative stress. Disturbances in the functioning of methionine sulfoxide reductase may have a significant impact on the development of many diseases, including schizophrenia. Various genetic variations of methionine sulfoxide reductase have been demonstrated in schizophrenic patients. They may be associated with dopamine disorders and affect the effects of treatment. These genetic variations affect various traits related to brain function. Chronic stress is associated with overexpression of methionine sulfoxide reductase in the hippocampus [161].
	*S*-carboxymethyl-cysteine *S*-2-succinyl-cysteine	Under the influence of glyoxal or glucose on cysteine residues, protective amino acids, peptides containing thiol groups, and proteins form S-carboxymethyl-cysteine, which has been recognized as AGE. Studies show that thiohemiacetal that was initially formed undergoes an intramolecular Cannizzarro reaction [162]. S-succinyl-cysteine is formed as a result of a nonenzymatic Michael reaction under the influence of fumaric acid on the free thiol groups of cysteine residues [157,163].	Fumarate, leading to the formation of S-(2-succinyl-cysteine, causes irreversible inhibition of many sulfhydryl enzymes. One of them is 3-phosphoglyceroldehyde dehydrogenase, which belongs to the glycolytic pathway, which is impaired in the course of schizophrenia [164].
	Carboxymethyllysine	Formed as a result of various reaction mechanisms, i.e., oxidation of fructosyl-lysine (reaction chain leading to AGE), direct reaction of glyoxal with the ε-amino group of lysine (reaction chain leading to ALE) [162,165], oxidation of lysine, proline residues, arginine and threonine [130].	Important AGE-epitope and RAGE ligand. It causes systemic glycoxidant load and increased body’s susceptibility to stress [165].
	Cysteine/cystine Homocysteine/homocystine	Homocysteine and cysteine are reduced forms, homocystine and cystine are oxidized forms [166]. Homocysteine is a product of metabolism (demethylation) of methionine. It is sensitive to autoxidation and can be converted into cysteine [167].	The accumulation of homocysteine and the lack of further metabolism of this compound causes a disorder of thymidine synthesis, DNA replication and neurogenesis, as well as the synthesis of neurotransmitters leading to a disorder of brain conductance [168]. Cysteine, which is a precursor of antioxidative glutathione, exhibits elevated levels in schizophrenia, compensating for the increase in oxidative stress [169].
Lipids	Lipid peroxides	Formed during oxidation of polyunsaturated long-chain fatty acids, e.g., linoleic, arachidonic, and docosahexaenoic acids. Hydrogen from the methyl group is removed first, resulting in the formation of two double bonds. Then, the fat is isomerized, a diene is formed, which reacts with molecular oxygen, leading to the formation of lipid peroxide. The second mechanism is based on oxidation catalyzed by metals (iron, copper). As a result of the Fenton reaction, radicals are formed that remove hydrogen from the methyl group, further reaction proceeds as described above [170].	Inhibits glycolysis and synthesis of proteins and nucleic acids. Leads to the disorders in the transport of glucose and glutathione, damaging cholinergic neurons and accelerating apoptosis of neurons. Binds to thiol groups of proteins or to glutathione, reducing their level in the cell; binds to amino groups of proteins or DNA nitrogen bases, initiating mutagenesis and carcinogenesis processes. The concentration in schizophrenic patients is higher in both the brain and the periphery [171,172]. They probably inhibit Na, K-ATPase activity by lipid peroxidation, which leads to disruption of the phospholipid moiety [173].
	Malondialdehyde	MDA is formed during conversion of methyl linoleate to prostaglandin-like endoperoxide, which is considered a precursor of MDA under stress. Another mechanism is the production of hydrogen peroxide and β-cleavage of the fatty acid chain. Hydroperoxyaldehyde is then formed, from which MDA is generated as a result of β-scission or due to the reaction of the acrolein radical with a hydroxyl radical. MDA can also be formed during the enzymatic biosynthesis of thromboxane A_2_ [130,134,174].	Inhibits glycolysis and synthesis of proteins and nucleic acids. Leads to the disorders in the transport of glucose and glutathione, damaging cholinergic neurons and accelerating apoptosis of neurons. Binds to thiol groups of proteins or to glutathione, reducing their level in the cell; binds to amino groups of proteins or DNA nitrogen bases, initiating mutagenesis and carcinogenesis processes. The concentration in schizophrenic patients is higher in both the brain and the periphery [171,172].
	4-hydroxynonenal	Oxidation of polyunsaturated fatty acids, i.e., linoleic, linolenic, arachidonic, and docosahexaenoic acids, by lipid peroxides [175]. Generally formed from omega-6 acids via β-fragmentation of 15-hydroperoxyarachidonic acid and 13-hydroperoxylolenic acid [130,134].	HNE induces the formation of protein adducts, which then become the cause of a toxic neuronal disorder. Increases permeability of BBB and endothelium of blood vessels [175,176].
	F2-isoprostanes	Eicosanoids result from the peroxidation of long-chain polyunsaturated fatty acids (mainly omega-3 and omega-6), e.g., arachidonic acid by OH^•^ [130,134,177].	Highly reactive products formed by the metabolism of isoprostanes (isoketals and their protein adducts). Inhibits the activity of simpleasomes, contributing to neurodegeneration [178,179].
DNA	8-hydroxydeoxyguanosine	Formed during oxidation (hydroxylation of the C-8 position) of nucleotide guanine by OH^•^.	Leads to the destruction of DNA, which causes an increase in mutagenicity, cancer risk, and neurodegenerative diseases [177,180].
	Uracil, xanthine, oxanine	Exposure of DNA to RNS (N_2_O_3_—product of NO˙ auto-oxidation) causes deamination of bases and conversion of cytosine to uracil (2’-deoxyuridine), guanine to xanthine (2’-deoxyxanthosine), oxanine (2’-deoxyoxanine) (in the presence of HNO_2_ at acidic pH), and 8-nitroguanine [181,182].	The correct level of uracil incision, abasic site cleavage, and dNTP incorporation activities in mitochondria originating from brains of patients with AD was examined [183]. Reduced activity of xanthine oxidase in the thalamus and occipital cortex of patients with chronic schizophrenia was observed. Studies show that elevated levels of xanthine oxidase also occur in the blood of schizophrenic patients [184].
	8-nitroguanine	˙NO_2_ and NOO^-^ react with guanine contained in DNA bases, nucleosides, and nucleotides, resulting in the formation of 8-nitroguanine [185,186].	The presence of 8-nitroguanine has been shown in hepatocytes, Kuppfer’s cells, and inflammatory cells of patients with chronic hepatitis C [187]. 8-Nitroguanine can lead to the formation of tumors and is detected in cancer cells [188,189].
	5-chlorocytosine	Formed under the direct influence of HClO on cytosine contained in DNA [190].	5-Chlorocytosine may serve as a biomarker for chronic inflammation. The presence of 5-chlorothyrosine predisposes to mutagenesis [191].
	5-chlorouracil	Formed due to the enzymatic deamination of 5-chlorocytosine [190].	Exhibits genotoxic and antimitotic effects [192].
	5-hydroxymethyluracil 5-hydroxyuracil	Formed under the influence of ROO^•^ [191]. Oxidation of cytosine to unstable cytosine glycol which undergoes deamination leads to the formation of 5-hydroxyuracil [193]. 5-hydroxymethyluracil can be formed in two mechanisms: Oxidation of thymine and a formation of the base with adenine; Formation under the influence of ROS, for example H_2_O_2_ with 5-methylcytosine. Then, 5-hydroxymethylcytosine is formed, which undergoes deamination to 5-hydroxymethyluracil, forming a base pair with cytosine [194].	Elevated levels of 5-hydroxyuracil have been observed in vulnerable regions of the brains of patients in the late stages of Alzheimer’s disease [195]. Elevated levels of 5-hydroxyuracil have also been observed in other brain regions (such as temporal, parietal, and frontal lobes) with AD. Elevated levels of 5-hydroxyuracil in mtDNA were examined in the parietal and temporal lobes of AD brains, while neocortex showed an increased content of 5-hydroxyuracil in nuclear DNA [196]. A reduced level of incision and 5-hydroxyuracil ligase activity in mitochondria from brain postmortem investigations was demonstrated in patients with AD [183]. 5-Hydroxymethyluracil plays an important role in DNA demethylation. It can serve as a cancer biomarker [197].
	Etheno-DNA-adducts	Reactive compounds formed in ROS-induced modification reactions, e.g., modified lipids, react with DNA either directly or through bi-functional intermediates, creating mutagenic etheno-DNA adducts, e.g., γ-linolenic acid peroxidation products as 4-hydroxynenenal react with adenine, cytosine, and guanine. This results in the formation of 1,N^6^-etheno-2’-deoxyadenosine (εdA), 3,*N*^4^-etheno-2’-deoxycytidine (εdC), 1,*N*^2^-etheno-2’-deoxyguanosine (1,N^2^εdG), and N^2^,3-etheno-2’-deoxyguanosine (N^2^,3εdG) [198].	Can be used as cancer markers [199]. They are carcinogenic and play an important role in initiating the process of hepatocarcinogenesis [200]. They are detected in the livers of patients with nonalcoholic steatohepatitis (NASH) [201] and alcoholic liver disease (ALD) [200].
RNA	8-hydroxyguanosine	Hydroxylation of nucleotide guanine by OH^•^, H_2_O_2_, O_2_^•−^ [202].	Increased serum 8-hydroxyguanosine levels have been demonstrated in patients with traumatic brain injury, which correlated with mortality [201]. Its occurrence is associated with many chronic diseases in old age, including Alzheimer’s disease [200], dementia and Parkinson’s disease [201].

**Table 3:** Total antioxidant capacity with regards to the key reactions for their determination.

Oxidative Potential Found in Literature	Key Reaction	References
TAC (total antioxidant capacity)	$AN=NA\to \left[A\cdot N_{2}\cdot A\right]\overset{-N_{2}}{\to }2A\cdot$ $A\cdot +O_{2}\to AO_{2}\cdot \overset{R-PE}{\to }stable\;product$ $AO_{2}\cdot +AO_{2}\cdot \to stable\;product$ R-PE: R-phycoerythrin	[233,237,238,239]
TAP/TAOP (total antioxidant power/potential)	Assessed via FRAP method: Fe^III^TPTZ + antioxidant → Fe^II^-TPTZ	[230,240,241]
TRAP (total radical-trapping antioxidant parameter)	$RN=NR\overset{37{}^{\circ}C}{\to }\left[R\cdot N_{2}\cdot R\right]\overset{-N_{2}}{\to }2R\cdot \overset{2O_{2}}{\to }2ROO\cdot$ RN=NR (ABAP)	[232,236,242]
TRAP (total reactive antioxidant potential)	Assessed via the method proposed by Lissi et al. $LH_{2}\overset{-H}{\to }LH\cdot \overset{O_{2}}{\to }LO_{2}H\cdot ⇋LHOOH$ ↘ ↙ hν $ROO\cdot +LH_{2}\to ROOH+LH\cdot$	[232,243]
TAR (total antioxidant reactivity)	Assessed using the abovementioned method	[228]
TAR (total antioxidant response)	Based on the method proposed by Miller et al. ABTS - 2,2′-azinobis-(3-ethylbenzothiazoline)-6-sulphonic acid $ABTS\overset{peroxidase\;\left(e.g.\;metmyoglobin\right),H_{2}O_{2}}{\to }ABTS^{\cdot +}$	[228,240]
TAA (total antioxidant activity)	$\mathrm{Fe}-\mathrm{EDTA}+H_{2}O_{2}\to OH\cdot \overset{bsodium\;benzoate}{\to }TBARS\;\overset{antioxidants}{\to }\;TBARS\;suppression$	[240,244]
TAS (total antioxidant status)	ABTS - 2,2′-azinobis-(3-ethylbenzothiazoline)-6-sulphonic acid $ABTS\overset{peroxidase\;\left(e.g.\;metmyoglobin\right),H_{2}O_{2}}{\to }ABTS^{\cdot +}$	[228,245,246]
TPAC (total plasma antioxidant capacity/capability)	$AN=NA\to \left[A\cdot N_{2}\cdot A\right]\overset{-N_{2}}{\to }2A\cdot$ $A\cdot +O_{2}\to AO_{2}\cdot \overset{R-PE}{\to }stable\;product$ $AO_{2}\cdot +AO_{2}\cdot \to stable\;product$ R-PE: R-phycoerythrin	[237,238,247]
NEAC (nonenzymatic antioxidant capacity)	$AN=NA\to \left[A\cdot N_{2}\cdot A\right]\overset{-N_{2}}{\to }2A\cdot$ $A\cdot +O_{2}\to AO_{2}\cdot \overset{R-PE}{\to }stable\;product$ $AO_{2}\cdot +AO_{2}\cdot \to stable\;product$ R-PE: R-phycoerythrin	[134,238]
Ir-reducing capacity (iridium-reducing capacity)	$Ir\left(IV\right)+e^{-}\to Ir\left(III\right)$ $Ir^{OX}+e^{-}\to Ir^{RED}$ $K_{2}IrCl_{6}\left(IV\right)+e^{-}\to Ir\left(III\right)$	[128,248]
